# NNDSS Tables Have Updated ‘N’ Indicators for 2011–2013

**Published:** 2013-03-29

Because of delays in the collection, processing, and analysis of data from the 2011 Council of State and Territorial Epidemiologists (CSTE) State Reportable Conditions Assessment (SRCA), 2011 SRCA data could not be applied to the National Notifiable Diseases Surveillance System (NNDSS) data in the *MMWR* NNDSS Tables I and II for 2011 or 2012. CSTE performed a major redesign of the SRCA application in 2011, which delayed CSTE’s data collection efforts. Additionally, CDC’s data quality control and assessment efforts and analysis of 2011 SRCA data were prolonged, in part because of challenges experienced in analyzing the data.

SRCA data are analyzed annually to create “not reportable” (“N”) indicators for *MMWR* Tables I and II, to denote when a nationally notifiable disease is not reportable in a specific reporting jurisdiction. As a temporary measure to update the “N” indicators applied to *MMWR* NNDSS Tables I and II, reporting jurisdictions informed CDC of which nationally notifiable infectious conditions were not reportable in their jurisdictions for years 2011 and again for year 2012. Thus, the *MMWR* NNDSS Tables I and II use 2011 and 2012 “N” indicators reported directly to CDC and not from the CSTE’s SRCA. The 2012 “N” indicators from CDC are being used for both the 2012 and 2013 NNDSS data. The 2011 “N” indicators from CDC are being used for the 2011 NNDSS data. NNDSS reporting exceptions (“N” indicators) for 2006 through 2012 can be found at http://wwwn.cdc.gov/nndss/script/downloads.aspx.

SRCA data CSTE currently displays on its web query page (http://www.cste2.org/izenda/entrypage.aspx) reflect the State Reportable Conditions Assessment results from 2007 through 2010 only. SRCA results for 2011 will be available on CSTE’s web site via a new web query tool being developed by CSTE.

